# Retracted: PKC*δ* Promotes High Glucose Induced Renal Tubular Oxidative Damage via Regulating Activation and Translocation of p66Shc

**DOI:** 10.1155/2022/9826078

**Published:** 2022-02-23

**Authors:** Oxidative Medicine and Cellular Longevity

Oxidative Medicine and Cellular Longevity has retracted the article titled “PKC*δ* Promotes High Glucose Induced Renal Tubular Oxidative Damage via Regulating Activation and Translocation of p66Shc” [1] due to concerns with the reliability of the data as initially raised by the authors.

A figure duplication has been identified in the LG/p-PKC*δ* and HG + Rottlerin/p-p66Shc panels of Figure 4(c). The authors explained that this error was introduced during the preparation of the manuscript.

Due to the above error, the authors reviewed the article and additionally identified that the semi-quantitative results presented in Figure 7(b) do not appear to correspond with the data shown in Figure 7(a). The data is no longer available to the authors for verification and the article is therefore retracted from the journal due the above concerns.

The authors agree to the retraction and the notice.
